# DOACs or VKAs or LMWH – What is the optimal regimen for cancer-associated venous thromboembolism? A systematic review and meta-analysis

**DOI:** 10.1016/j.amsu.2022.103925

**Published:** 2022-06-09

**Authors:** Naser Yamani, Samuel Unzek, Talal Almas, Adeena Musheer, Arooba Ejaz, Anousheh Awais Paracha, Izza Shahid, Farouk Mookadam

**Affiliations:** a Department of Medicine, John H Stroger Jr. Hospital of Cook County, Chicago, IL, USA; b Department of Cardiac Imaging, Banner University Medical Centre, Scottsdale, AZ, USA; c Department of Medicine, RCSI University of Medicine and Health Sciences, Dublin, Ireland; d Department of Medicine, Dow University of Health Sciences, Karachi, Pakistan; e Department of Medicine, Ziauddin Medical University, Karachi, Pakistan; f Department of Cardio Oncology, Banner University Medical Centre, Phoenix, AZ, USA

**Keywords:** Anticoagulant, Cancer, Mortality, Embolism, Bleeding

## Abstract

**Background:**

Clinical guidelines have supported the use of direct anticoagulants (DOACs) for the treatment of cancer-associated venous thromboembolism (Ca-VTE). However, recent trials have reported increased bleeding risks associated with DOACs usage, raising concerns regarding its efficacy.

**Objectives:**

The authors conducted a meta-analysis to study the efficacy and safety of DOACs for the treatment of VTE in cancer patients, compared with Low-weight molecular heparin (LMWH) and Vitamin-K antagonists (VKAs).

**Methods:**

PubMed, EMBASE, Cochrane Library, Cochrane Central Register of Controlled Trials (CENTRAL) were searched according to the Preferred Reporting Items for Systematic Reviews and Meta-Analyses (PRISMA) guidelines from inception to June 17th, 2021.The primary outcomes studied were VTE recurrence and major bleeding.

**Results:**

A total of 8 randomized controlled trials (RCTs) enrolling almost 7000 patients were included. Direct oral anticoagulants significantly reduced VTE Recurrence in cancer patients when compared to patients treated with LMWH or VKAs (Hazard ratio [HR] 0.62, 95% confidence interval [CI] 0.46–0.83; P = 0.002; I^2^ = 26%). There were no statistically significant differences for major bleeding (HR 0.86, 95% confidence interval [CI] 0.56–1.33; P = 0.50; I^2^ = 34%), clinically relevant non-major bleeding (HR 1.23, 95% confidence interval [CI] 0.79–1.91; P = 0.35; I^2^ = 66%), pulmonary embolism (HR 0.71, 95% confidence interval [CI] 0.47–1.06; P = 0.10; I^2^ = 7%), and all-cause mortality (HR 0.98, 95% confidence interval [CI] 0.86–1.12; P = 0.78; I^2^ = 1%), between DOACs and LMWH.

**Conclusion:**

This analysis shows that DOACs are the optimal regimen to treat Ca-VTE. They have a similar to slightly increased bleeding risk compared with LMWH and are a safer alternative to VKAs.

## Highlights

• Patients receiving DOACs show a significant decrease for VTE recurrence.
• Rivaroxaban pertaining to the most effective DOAC.
• Edoxaban proving to be more useful in reducing bleeding risk more than LMWHs.
• Further research is needed to investigate the correlation between cancer types and the efficacy of DOACs in treating Ca-VTE.

## Graphical abstract

**Figure:**
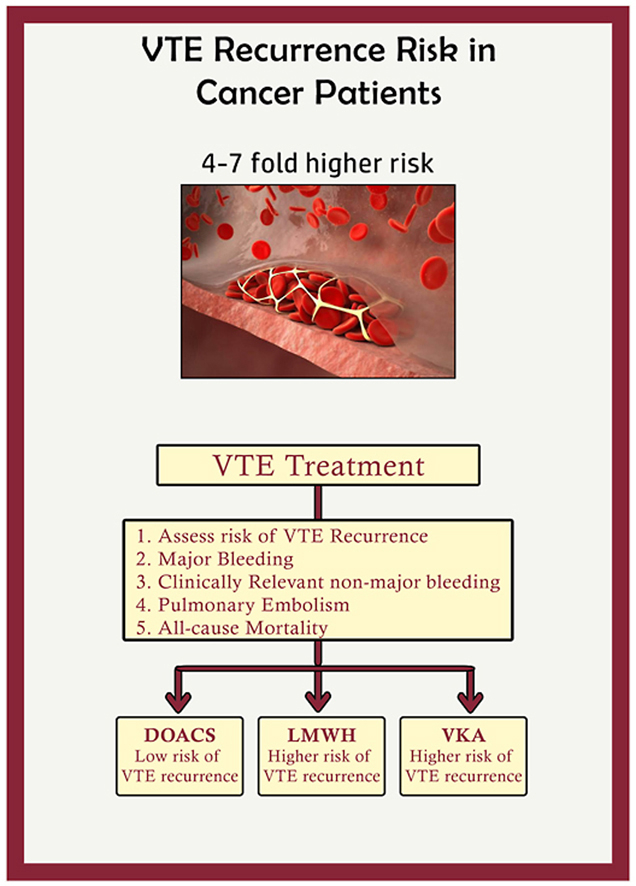

## Abbreviations

DOAC: Direct Oral Anticoagulants VKA: Vitamin K Antagonist
LMWH: Low Molecular Weight Heparin VTE: Venous Thromboembolism
DVT: Deep Vein Thrombosis PE: Pulmonary Embolism
CRNMB: Clinically relevant non-major Bleeding
Ca-VTE: Cancer-associated Venous Thromboembolism RCT: Randomized Clinical Trials

## 1 Introduction

Venous thromboembolism (VTE) is associated with increased morbidity and mortality in cancer patients [1]. In comparison to non-cancer patients, VTE recurrence has a 4–7fold higher incidence in patients with cancer [2]. The occurrence of VTE is commonly in the form of deep vein thrombosis (DVT) and pulmonary embolism (PE) [3]. In order to prevent VTE recurrence, anticoagulant therapy is recommended. However, the concomitant bleeding risk makes the management of VTE in patients challenging. Hence, it is crucial to find an anticoagulant regimen that maintains an ideal balance between efficacy and safety outcomes of VTE recurrence and bleeding, respectively.

Clinical guidelines have upheld the conventional use of low-weight molecular heparin (LMWH) as standard of care and optional use of Vitamin-K Antagonists (VKA) for the treatment of cancer-associated VTE (Ca-VTE) [4]. However, direct oral anticoagulants (DOACs), including rivaroxaban, dabigatran, edoxaban and apixaban, are recently gaining recognition as viable alternatives to LMWH and VKAs for reducing VTE events in cancer patients. According to guidelines from the International Society on Thrombosis and Haemostasis (ISTH), DOACs can be used for the treatment of VTE in cancer patients [5]. At the same time, recent studies have shown that while DOACs have a similar risk for VTE recurrence compared to LMWH, the use of DOACs was associated with increased rates of clinically relevant bleeding [6,7]. Nevertheless, there is a lack of pooled data and systematic reviews that investigate the safety and efficacy of DOACs when compared to LMWH and VKAs for the treatment of VTE in cancer patients.

For the purpose of clarifying the effectiveness and safety of DOACs, we conducted a systematic review and meta-analysis, along with subgroup analyses of cancer types and types of DOACs and their association to primary efficacy outcome of VTE recurrence.

## 2 Methods

### 2.1 Data sources and search strategy

This meta-analysis was conducted according to the Preferred Reporting Items for Systematic Reviews and Meta-Analyses (PRISMA) guidelines [8,24]. The literature from the PubMed, EMBASE, Cochrane Library, Cochrane Central Register of Controlled Trials (CENTRAL), and ClinicalTrials.gov databases with a publication date from inception to June 17th, 2021 was systematically searched by two independent reviewers (AM and AE) using an extensive search strategy in the Supplemental Table 1. No IRB approval was required as this research is a meta-analysis.

### 2.2 Study selection

Articles were independently reviewed for inclusion, and any discrepancies between reviewers (AM and AE) were discussed and resolved with a senior investigator (NY). Articles that met the following criteria were included; were [1] Randomized Controlled Trials had [2] Adult Patients >18 years of age [3] Patients with VTE and Cancer (Active or History of Cancer).

[4] with follow-up time of minimum 6 months [5] compared DOACs to LMWH alone, VKAs alone, LMWH followed by VKAs, or VKAs followed by LMWH [6] reported VTE Recurrence as the Efficacy Outcome and Major Bleeding as the Safety Outcome [7] reported at least any three of these outcomes; Pulmonary Embolism, clinically relevant non-major Bleeding or All-cause Mortality as Secondary Outcomes.

### 2.3 Data extraction

Studies were selected by 2 reviewers (AM and AE) independently, compiled in Endnote Reference Library (Version X7.5; Clarivate Analytics, Philadelphia, Pennsylvania) software where duplicates were searched and removed, and results were compared; any discrepancies were further discussed with other authors to achieve full consensus. After a full-text review of 24 studies, 8 RCTs were included. Study extraction results are presented in the 2009 Prisma Flow Diagram (Supplemental Table 1). The remaining studies were excluded due to underlying conditions such as Atrial Fibrillation and prophylactic treatments. We extracted the following information from each study: participants’ sample size, sex, cancer status (active cancer or history of cancer), type of cancers and associated regimen, interventions, outcomes, duration of follow-up and bleeding risk (Table 1).

**Table 1:** Study characteristics and outcomes of the included studies.

Characteristic	Prins, 2014	Agnelli, 2015	Schulman, 2015	Raskob, 2016	Young, 2018	Raskob, 2018	McBane, 2020	Agnelli, 2020
Trial name	Oral rivaroxaban versus enoxaparin with vitamin K antagonist for the treatment of symptomatic venous thromboembolism in patients with cancer (EINSTEIN-DVT and EINSTEIN PE): a pooled subgroup analysis of two randomized controlled trials-	Oral apixaban for the treatment of venous thromboembolism in cancer patients: results from the AMPLIFY trial	Treatment with dabigatran or warfarin in patients with venous thromboembolism and cancer	Edoxaban for venous thromboembolism in patients with cancer: results from a non- inferiority subgroup analysis of the Hokusai- VTE randomized, double-blind, double-dummy trial	Comparison of an Oral Factor Xa Inhibitor with Low Molecular Weight Heparin in Patients With Cancer With Venous Thromboembolism: Results of a Randomized Trial (SELECT-D)	Edoxaban for the Treatment of Cancer Associated Venous Thromboembolism	Apixaban and dalteparin in active malignancy- associated venous thromboembolism: The ADAM VTE trial	Apixaban for the Treatment of Venous Thromboembolism Associated with Cancer
Patients, n Enrollment initiation	1124	523	336	979	406	1046	287	1155
	March 22, 2007	July 2008	2006	Jan 28, 2010	September 6, 2013	July 2015	November 20, 2015	April 2017
Enrollmentcompletion Year of publication	March 12, 2011	March 2013	2010	Oct 31, 2012	December 22, 2016	December 2016	October 2, 2017	June 2019
	2014	2015	2015	2016	2018	2018	2020	2020
Trial type	subgroup analysis of patients with cancer enrolled in the EINSTEINDVT and EINSTEIN-PEopen-label, phase3, randomizedcontrolled trials.l	subgroup analysis of patients with cancer on VTE treatment enrolled in the AMPLIFY was a randomized,double-blind trial	post-hoc analysisof CA-VTEpatients enrolled in RECOVER and RECOVER II; both studies were randomized, double-blind, double-dummy trials	post-hoc analysisof patients with cancer enrolled in Hokusai-VTE trial; Hokusai- VTE was a multicenter randomized, double-blind, double-dummy trail	randomized, open-label, multicenter pilot trial	randomized, open-label trial	randomized, open-label, investigator-initiated trial	multinational, randomized, investigator-initiated, open-label,noninferiority trial
Randomization sequence	computerized voice-response system	interactive voice- response system	interactive voice response system	interactive, web- based system	central randomization, computer-generated	interactive, web- based system	interactive, web- based system	interactive, online system
Treatments	DOAC (rivaroxaban were given 15 mg orally twice daily for 21 days, followed by 20 mg/d)LMWH followed by VKA (enoxaparin 1.0 mg/kg/d; warfarin INR 2.0–3.0)	DOAC apixaban(10 mg twice daily for 7 days followed by 5 mg twice daily) LMWH followed by VKA (enoxaparin 1 mg/kg twice daily for at least 5 days, followed by dose- adjusted warfarin)	DOAC(dabigatran was given 150 mg twice daily)VKA (WarfarinINR (2.0–3.0))	DOAC (edoxaban60mg/day)VKA (WarfarinINR (2.0–3.0))	DOAC(rivaroxaban were given 15 mg twice/day for 30 days, followed given 20 mg/day)LMWH (dalteparin 200 IU/kg daily for 1 month, followed by 150 IU/kg daily	LMWH followed by DOAC (heparin for at least 5 days followed by oral edoxaban at a dose of 60 mg once daily) LMWH (subcutaneous dalteparin at a dose of 200 IU/kg once daily for 1 month followed by dalteparin at a dose of 150 IU/kg once daily	DOAC (apixaban 10 mg twice daily for seven days, followed by 5 mg/day) LMWH (dalteparin 200 IU/kg daily for one month, followed by 150 IU/kg daily)	DOAC (apixaban 10 mg twice daily for seven days, followed by 5 mg/day) LMWH (dalteparin 200 IU/kg daily for one month, followed by 150 IU/kg daily)
Definition of primary efficacy outcome	recurrent venous thromboembolism	recurrent venous thromboembolism	recurrent venous thromboembolism	recurrent venous thromboembolism	recurrent venous thromboembolism	recurrent venous thromboembolism	any thromboembolic recurrence, including venous thromboembolism, DVT, PE	recurrent venous thromboembolism
Active or history of cancer	Either active cancer or history of cancer	Either active cancer or history of cancer	Active cancer	Either active cancer or history of cancer	Active cancer	Either active canceror history of cancer	Active cancer	Either active canceror history of cancer
Follow-up	12 months	6 months	6 months	3–12 months	12 months	12 months	6 months	6 months

VTE: Venous Thromboembolism, MB: Major Bleeding, CRNMB: Clinically Relevant non-major Bleeding, DVT: Deep vein thrombosis, PE: Pulmonary embolism, DOAC: Direct oral anticoagulant, VKA: Vitamin K Antagonist, LMWH: Low-molecular-weight Heparin.

### 2.4 Data analysis

**Fig. 1:**
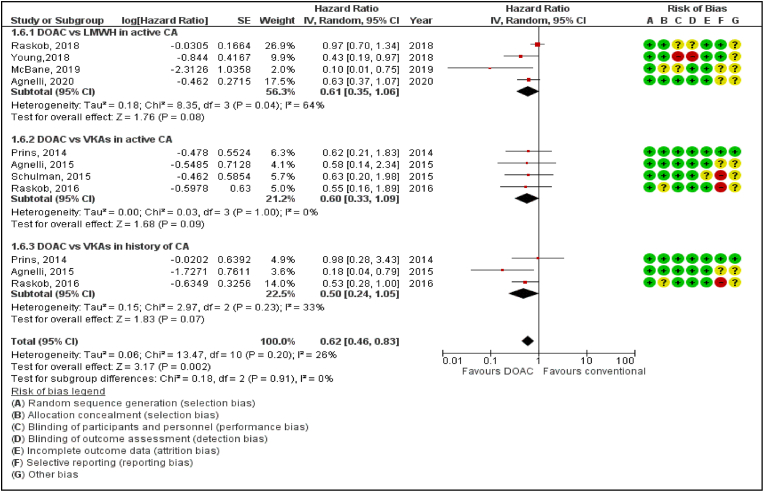
Forest plot displaying the effect of DOAC and LMWH or VKAs on Major Bleeding in Cancer Patients.

Statistical Analysis was performed by extracting Hazard Ratios (HR) and corresponding 95% CIs from each trial for primary(VTE Recurrence and Major Bleeding) and secondary outcomes(CRNMB, PE and All-cause mortality). Data was pooled using the Inverse Variance method and random-effects model in the Cochrane Review Manager software (RevMan version 5.4.1). Heterogeneity between included studies was assessed by visual inspection of Forest plots and the I^2^ statistic, which examined the percentage of variation across studies caused by heterogeneity rather than chance. An I^2^ value of 0% indicated no heterogeneity, whereas larger values indicated increasing heterogeneity. The association of risk of VTE recurrence between patients with active cancer or a prior history of cancer were identified and analysed in our study using Hazard Ratios (HR) (Fig. 1). In addition, sub-group analyses was performed to evaluate whether the efficacy of DOAC vs Conventional Therapy is affected by the type of cancer (Supplementary Figure 5). This meta-analysis also includes a sub-group analysis of the type of DOAC used and its association with the overall efficacy of DOAC vs Conventional Therapy (Supplementary Fig. 3A and 3B). P-value < 0.05 was considered significant for all the above analyses.

### 2.5 Quality assessment

To assess the quality of the 8 RCTs across six domains (selection bias, performance bias, detection bias, attrition bias, reporting bias, and other biases), Cochrane Collaboration's risk of bias tool was used by 2 reviewers independently according to recommendations outlined in the Cochrane Handbook for Systematic Reviews of Interventions [9]. The quality of this systematic review was evaluated using the AMSTAR-2 criteria. Our systematic review is partially compliant with the AMSTAR-2 criteria as indicated in the AMSTAR-2 checklist [25].

## 3 Results

### 3.1 Study characteristics

Our initial search yielded 2216 studies, of which 24 were selected for full-text review.

After exclusions, eight studies with approximately 7000 patients were included in the final analysis.

The baseline characteristics are provided in Supplemental Table 1. Among the included studies, four compared DOACs with LMWH, and four studies investigated the use of DOACs versus VKAs. In terms of DOACs, three studies were designed using apixaban, two using rivaroxaban, two using edoxaban, and one using dabigatran. Vitamin K Antagonists used, were warfarin and acenocoumarol. Low Molecular Weight Heparins used included enoxaparin and dalteparin. Of the DOAC versus VKA studies, three reported separate values for patients with active and a history of cancer for efficacy and safety outcomes, while one included patient with active cancer only. Four studies comparing DOACs and LMWH comprised a patient population with the majority being active cancer patients, and one study included patients with both active cancer and a history of cancer. Patients with various types of cancer, including breast, lung, gastrointestinal, brain, pancreatic, melanoma, sarcoma, and genitourinary, were investigated in these studies. The follow-up period ranged from 3 to 12 months. According to the Cochrane Risk of Bias Assessment, most studies reported a low risk of bias.

## 4 Outcomes

### 4.1 VTE recurrence

All the RCTs included in this meta-analysis report VTE Recurrence as their primary efficacy outcome. Data pooled to identify the optimal regimen to treat VTE in patients was further divided into subgroups of patients with active or history of cancer. Direct oral anticoagulants significantly reduced VTE Recurrence in cancer patients when compared to patients treated with LMWH or VKAs. (Hazard ratio [HR] 0.62, 95% confidence interval [CI] 0.46–0.83; P = 0.002; I^2^ = 26%) (*P*‐value for subgroup differences = 0.91). (Fig. 1).

### 4.2 Major bleeding

**Fig. 2:**
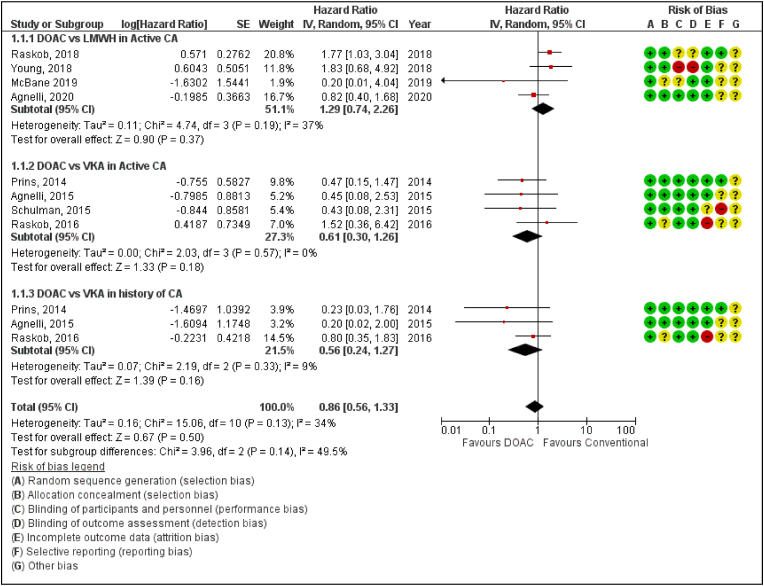
Forest plot displaying the effect of DOAC and LMWH or VKAs on Major Bleeding in Cancer Patients. IV: Inverse variance; CI: Confidence interval; SE: Standard error.

DOACs were generally associated with a lower risk of bleeding than VKAs and LMWH (Hazard ratio [HR] 0.86, 95% confidence interval [CI] 0.56–1.33; P = 0.50; I^2^ = 34%) (*P*‐value for subgroup differences = 0.14). (Fig. 2). A better safety profile is seen with the use of DOAC compared to VKA as shown in Fig. 2. Even though with DOACs there were a relatively greater number of major bleeding events compared to LMWH in patients with active cancer, there is no significant difference between the two. (Hazard ratio [HR] 1.29, 95% confidence interval [CI] 0.74–2.26; P = 0.37; I^2^ = 37%)(*P*‐value for subgroup differences = 0.14).

### 4.3 Clinically Relevant Non-Major Bleeding (CRNMB)

**Fig. 3:**
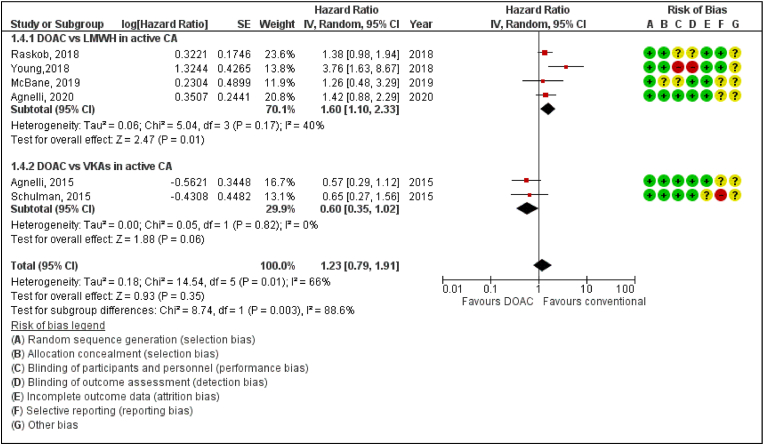
Forest plot displaying the effect of DOAC and LMWH or VKAs on CRNMB in Cancer Patients. IV: Inverse variance; CI: Confidence interval; SE: Standard error.

In the sub-group analysis of DOAC vs LMWH and DOAC vs VKA, pooled data shows that DOAC is associated with a statistically significant higher risk of CRNMB than LMWH(Hazard ratio [HR] 1.60, 95% confidence interval [CI] 1.10–2.33; P = 0.01; I^2^ = 40%) but a lower risk of CRNMB compared to VKAs (Hazard ratio [HR] 0.60, 95% confidence interval [CI] 0.35–1.02; P = 0.06; I^2^ = 0%). The overall effect size was reported as (Hazard ratio [HR] 1.23, 95% confidence interval [CI] 0.79–1.91; P = 0.35; I^2^ = 66%) and (*P*‐value for subgroup differences = 0.003) therefore statistically nonsignificant results are reported for DOAC vs Conventional Therapy (Fig. 3).

### 4.4 Pulmonary embolism (PE)

**Fig. 4:**
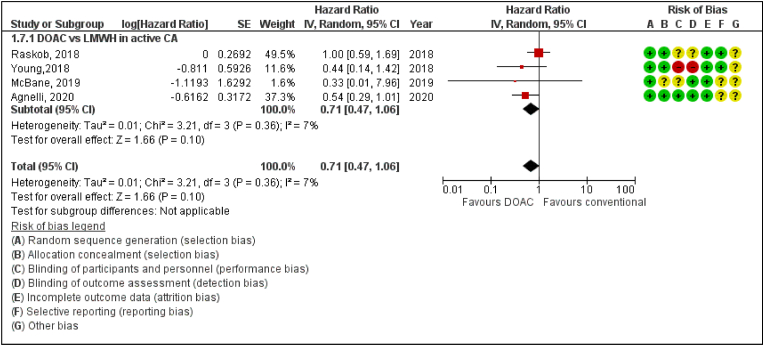
Forest plot displaying the effect of DOAC versus LMWH on Pulmonary Embolism in Cancer Patients. IV: Inverse variance; CI: Confidence interval; SE: Standard error.

The DOAC therapy does reduce the incidence of PE in patients with active cancer compared to LMWH but there was borderline significant difference between the DOAC and LMWH groups in the incidence of pulmonary embolism amongst cancer patients (Hazard ratio [HR] 0.71, 95% confidence interval [CI] 0.47–1.06; P = 0.10; I^2^ = 7%) (Fig. 4).

### 4.5 All-cause mortality

**Fig. 5:**
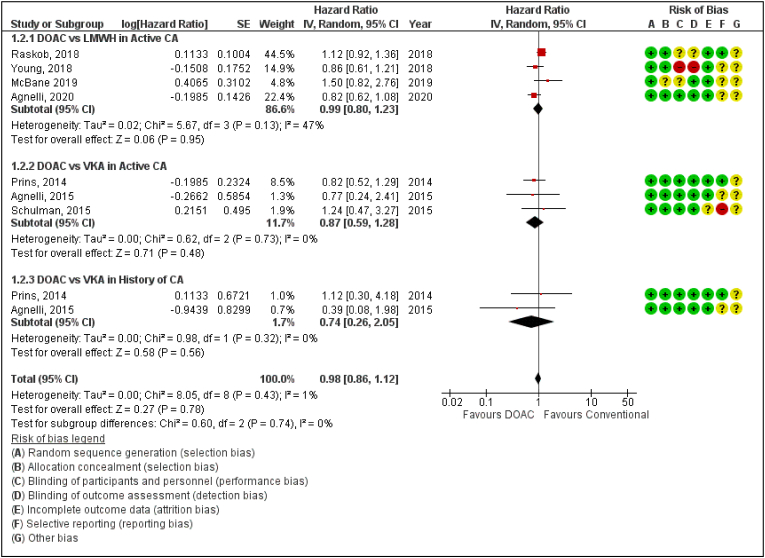
Forest plot displaying the effect of DOAC and LMWH or VKAs on All-cause Mortality in Cancer Patients. IV: Inverse variance; CI: Confidence interval; SE: Standard error.

Pooled analysis shows a lower rate of mortality in patients treated with DOACs compared to conventional therapy of either VKA or LMWH, however, no statistically significant difference was found between the two groups of cancer patients. The overall effect size was reported as (Hazard ratio [HR] 0.98, 95% confidence interval [CI] 0.86–1.12; P = 0.78; I^2^ = 1%) and (*P*‐value for subgroup differences = 0.74) (Fig. 5).

### 4.6 Subgroup analysis

The forest plots displaying subgroup analyses are present in Supplementary Material. For primary efficacy and safety outcomes, no subgroup difference was observed upon stratification according to type of DOAC (P-values for subgroup differences = 0.20 and 0.11, respectively).

Furthermore, the subgroup analysis performed to analyze possible relation between VTE recurrence and type of cancer, revealed no significant association (P-value for subgroup differences = 0.13).

## 5 Discussion

In this extensive meta-analysis comprising approximately 7000 cancer patients, DOACs significantly reduce the risk of VTE and DVT as compared to LMWH and VKAs. These results were consistent when patients were further stratified according to type of DOAC for both primary and secondary safety outcomes. There was no statistically significant difference in effect on risks for major bleeding, clinically relevant bleeding, and mortality between the two groups. However, sensitivity analysis (Fig. 5 in Supplementary Material) for CRNMB revealed that DOACs significantly reduce the risk of CRNMB as compared to LMWH.

In our meta-analysis, we report major findings for Ca-VTE Recurrence, major bleeding, pulmonary embolism, CRNMB and all-cause mortality in a cohort of almost 7000 patients treated with DOAC and VKAs or LMWH. Firstly, DOAC compared with conventional therapy significantly reduces the recurrence of VTE in cancer patients. (Fig. 1). Secondly, DOACs have also shown borderline significance in reducing the risk of developing PE as well as reducing mortality rates amongst all cancer patients (Fig. 4, Fig. 5).

According to the National Comprehensive Cancer Network's [10] and The International Initiative on Thrombosis and Cancer's [11] recent guidelines, DOACs have been included in the preferred list of regimens used to treat Ca-VTE. The powered RCTs included in this Meta- Analysis and their pooled results further reinforce the effectiveness of DOACs. The pooled subgroup analysis of EINSTEIN-PE and EINSTEIN-DVT [12], and the SELECT-D trial [13] observed the lowest rate of Ca-VTE amongst patients treated with Rivaroxaban. For bleeding risk including Major Bleeding and CRNMB amongst cancer patients, LMWH was non inferior to DOACs, yet Edoxaban reduced the bleeding risk more than the LMWHs, Enoxaparin and Dalteparin, as observed in the post-hoc analysis of the HOKUSAI-Trial [14]) (Supplementary Figure 3B).

It is important to highlight that all RCTs included show a prominent reduction of mortality rates with the use of DOAC compared to conventional therapy amongst cancer patients along with [15] accentuating the efficacy of DOAC in largely reducing PE occurrence compared to LMWH.

Assessing the efficacy of DOAC against VKA shows lower CRNMB events for DOACs [16,17], overall effect of all included trials that compare DOAC to LMWH, show superiority of LMWH over DOAC for reducing CRNMB risk amongst cancer patients.

The sub-group analysis performed to analyze the association of the type of cancer and efficacy of DOACs (Supplementary Figure 4) shows that there was no influence of cancer type on the efficacy of DOACs. Although, there are insufficient number of studies reporting the types of cancer therefore this finding is subject to further investigation.

Our principal findings are consistent with previous meta-analyses which have pooled the same RCT data [18,19]. The recent Song et al. meta-analysis [20], however, differed from our current meta-analysis in significant aspects. For instance, there was no stratification of the patient population according to cancer status, regardless of the fact that cancer status has a crucial influence on the risk for VTE. In addition to this, we also ensured our secondary safety outcomes were holistic and inclusive, and hence included mortality outcomes. To avoid high levels of heterogeneity because of pooling studies with varying risk of bias, we excluded small studies and cohorts from our analysis. Furthermore, since Agnelli et al. [17] and Schulman et al. [16] reported outcomes that were contradictory to the other studies in the case of clinically relevant non-major bleeding, we carried out a sensitivity analysis, which in turn revealed that DOACs significantly increase the risk of clinically relevant non-major bleeding as compared to LMWH (HR: 1.60 (1.10, 2.33); P = 0.01) (Supplementary Figure 5). Moreover, in contrast to Song et al., we used a random-effects model after taking into consideration the expected methodological heterogeneities between studies due to design, outcome definitions and drug dosage [21]. We also performed a subgroup analysis of types of cancer, to investigate the effect of cancer type upon VTE recurrence and major bleeding outcomes.

### 5.1 Study limitations

There are a few limitations of this analysis. Studies with patients having other comorbidities such as Atrial Fibrillation and patients given prophylaxis, were excluded. We conducted a thorough and extensive literature review to retract all potential and powered RCTs focused only on Ca-VTE and its treatment to draw valid conclusions. Out of nine RCTs, two being an open-label trial adds to the selection bias, however the other seven RCTs being a close-label trial overpower that bias. There are 3 new randomized controlled trials that reached its completion [22,23], however its results have not been posted on clinicaltrials.gov yet.

This Meta-Analysis includes all the latest RCTs that were published in 2014, ahead of the most recent data for Ca-VTE and its treatment. Due to a relatively smaller sample size of the included RCTs, results from the CANVAS [22] and CASTA-DIVA [23] trials are eagerly awaited because of their larger sample size, 940 and 200, respectively.

Despite a random-effects model used to counter heterogeneity, some differences between studies can limit our findings. These include the use of different types of DOACs, VKAs and LMWH, different percentages of men and women, and variations in comorbidities and baseline therapy.

## 6 Conclusion

In conclusion, DOACs essentially reduce the risk of Ca-VTE with similar or slightly increased bleeding risk compared to LMWH. DOACS are a safer alternative to VKA. Our findings are a portrayal of DOACs as the optimal regimen to treat Ca-VTE and PE, and additionally show a promising decreasing effect in mortality, regardless of the cancer status in these patients.Provenance and peer review Not commissioned, externally peer-reviewed.

## Ethical approval

N/A.

## Please state any sources of funding for your research

None to declare.

## Author contribution

Naser Yamani and Samuel Unzek – Conceptualization and designing the study Adeena Musheer, Arooba Ejaz and Anousheh Awais Paracha - drafting of the manuscript and data collection. Izza Shahid and Talal Almas – careful analysis of the data and interpretation of results Farouk Mookadam – final review and approval for submission.

## Please state any conflicts of interest

None to declare.

## Registration of research studies

1. Name of the registry.

2. Unique Identifying number or registration ID.

3. Hyperlink to your specific registration (must be publicly accessible and will be checked)

## Guarantor

Talal Almas.

Talalalmas.almas@gmail.com.

## Consent

N/A.

## Conflicts of Interest

None to declare Funding.

## Source

None to declare.
