# Supplementary material for: DOACs or VKAs or LMWH – What is the optimal regimen for cancer-associated venous thromboembolism? A systematic review and meta-analysis
Source: Ann Med Surg (Lond). 2022 Jun 9;79:103925. doi: 10.1016/j.amsu.2022.103925 (PMC9289311; doi:10.1016/j.amsu.2022.103925)

# SUPPLEMENTAL APPENDIX

**Supplemental Table S1**. Search strategy used in each database searched

**Supplemental Figure 1**. Prisma Flow Diagram

**Supplemental Figure 2a**. Quality Assessment of Included Randomized Controlled Trials using Cochrane Risk of Bias Tool; Risk of Bias Graph

**Supplemental Figure 2b**. Quality Assessment of Included Randomized Controlled Trials using Cochrane Risk of Bias Tool; Risk of Bias Summary

**Supplemental Table S2.** Baseline Characteristics of Patients

**Supplemental Figure 3**. Forest displaying subgroup analysis according to type of DOAC for the following outcomes: (A) venous thromboembolism, (B) major bleeding

**Supplemental Figure 4**. Forest plot displaying subgroup analysis according to type of cancer for venous thromboembolism

**Supplemental Figure 5**. Forest displaying sensitivity analysis for clinically relevant non-major bleeding

**Supplemental Table 1**. Search strategy used in each database searched

| **Database (Articles**  **Retrieved)** | **Search Strategy** |
| --- | --- |
|  | ((("direct"[All Fields] OR "directed"[All Fields] OR "directing"[All Fields] OR "direction"[All Fields] OR "directional"[All Fields] OR "directions"[All Fields] OR "directivities"[All Fields] OR "directivity"[All Fields] OR "directs"[All Fields]) AND ("mouth"[MeSH Terms] OR "mouth"[All Fields] OR "oral"[All Fields]) AND ("anticoagulants"[Pharmacological Action] OR "anticoagulants"[MeSH Terms] OR "anticoagulants"[All Fields] OR "anticoagulant"[All Fields] OR "anticoagulate"[All Fields] OR "anticoagulated"[All Fields] OR "anticoagulating"[All Fields] OR "anticoagulation"[All Fields] OR "anticoagulations"[All Fields] OR "anticoagulative"[All Fields])) OR "DOAC"[All Fields] OR (("novel"[All Fields] OR "novel s"[All Fields] OR "novels"[All Fields]) AND ("mouth"[MeSH Terms] OR "mouth"[All Fields] OR "oral"[All Fields]) AND ("anticoagulants"[Pharmacological Action] OR "anticoagulants"[MeSH Terms] OR "anticoagulants"[All Fields] OR "anticoagulant"[All Fields] OR "anticoagulate"[All Fields] OR "anticoagulated"[All Fields] OR "anticoagulating"[All Fields] OR "anticoagulation"[All Fields] OR "anticoagulations"[All Fields] OR "anticoagulative"[All Fields])) OR ("New"[All Fields] AND ("mouth"[MeSH Terms] OR "mouth"[All Fields] OR "oral"[All Fields]) AND ("anticoagulants"[Pharmacological Action] OR "anticoagulants"[MeSH Terms] OR "anticoagulants"[All Fields] OR "anticoagulant"[All Fields] OR "anticoagulate"[All Fields] OR "anticoagulated"[All Fields] OR "anticoagulating"[All Fields] OR "anticoagulation"[All Fields] OR "anticoagulations"[All Fields] OR "anticoagulative"[All Fields])) OR ("n 4 oleylcytosine arabinoside"[Supplementary Concept] OR "n 4 oleylcytosine arabinoside"[All Fields] OR "noac"[All Fields]) OR ("Target-specific"[All Fields] AND ("mouth"[MeSH Terms] OR "mouth"[All Fields] OR "oral"[All Fields]) AND ("anticoagulants"[Pharmacological Action] OR "anticoagulants"[MeSH Terms] OR "anticoagulants"[All Fields] OR "anticoagulant"[All Fields] OR "anticoagulate"[All Fields] OR "anticoagulated"[All Fields] OR "anticoagulating"[All Fields] OR "anticoagulation"[All Fields] OR "anticoagulations"[All Fields] OR "anticoagulative"[All Fields])) OR ("2 2 6 6 tetramethylpiperidine n oxide 4 amino 4 carboxylic acid"[Supplementary Concept] OR "2 2 6 6 tetramethylpiperidine n oxide 4 amino 4 carboxylic acid"[All Fields] OR "toac"[All Fields]) OR ("factor xa inhibitors"[Pharmacological Action] OR "factor xa inhibitors"[MeSH Terms] OR ("factor"[All Fields] AND "xa"[All Fields] AND "inhibitors"[All Fields]) OR "factor xa inhibitors"[All Fields] OR ("direct"[All Fields] AND "factor"[All Fields] AND "xa"[All Fields] AND "inhibitors"[All Fields]) OR "direct factor xa inhibitors"[All Fields]) OR ("Non-Vitamin"[All Fields] AND "K"[All Fields] AND ("antagonist"[All Fields] OR "antagonists and inhibitors"[MeSH Subheading] OR ("antagonists"[All Fields] AND "inhibitors"[All Fields]) OR "antagonists and inhibitors"[All Fields] OR "antagonists"[All Fields])) OR "non-VKA"[All Fields] OR ("rivaroxaban"[MeSH Terms] OR "rivaroxaban"[All Fields]) OR ("apixaban"[Supplementary Concept] OR "apixaban"[All Fields] OR "apixaban s"[All Fields]) OR ("edoxaban"[Supplementary Concept] OR "edoxaban"[All Fields]) OR ("dabigatran"[MeSH Terms] OR "dabigatran"[All Fields] OR "dabigatran s"[All Fields]) OR ("betrixaban"[Supplementary Concept] OR "betrixaban"[All Fields]) OR (("vitamin k"[MeSH Terms] OR "vitamin k"[All Fields]) AND ("antagonist"[All Fields] OR "antagonists  and inhibitors"[MeSH Subheading] OR ("antagonists"[All Fields] AND "inhibitors"[All Fields]) OR "antagonists and inhibitors"[All Fields] OR |

| Cochrane Library (1587 results) | "antagonists"[All Fields])) OR "VKA"[All Fields] OR ("warfarin"[MeSH Terms] OR "warfarin"[All Fields] OR "warfarin s"[All Fields] OR "warfarinization"[All Fields] OR "warfarinized"[All Fields] OR "warfarins"[All Fields]) OR ("warfarin"[MeSH Terms] OR "warfarin"[All Fields] OR "coumadin"[All Fields] OR "warfarin s"[All Fields] OR "warfarinization"[All Fields] OR "warfarinized"[All Fields] OR "warfarins"[All Fields]) OR ("heparin"[MeSH Terms] OR "heparin"[All Fields] OR "heparine"[All Fields] OR "heparins"[All Fields] OR "heparin s"[All Fields] OR "heparinate"[All Fields] OR "heparinated"[All Fields] OR "heparines"[All Fields] OR "heparinic"[All Fields] OR "heparinisation"[All Fields] OR "heparinised"[All Fields] OR "heparinization"[All Fields] OR "heparinize"[All Fields] OR "heparinized"[All Fields] OR "heparinizing"[All Fields])) AND ("cancer s"[All Fields] OR "cancerated"[All Fields] OR "canceration"[All Fields] OR "cancerization"[All Fields] OR "cancerized"[All Fields] OR "cancerous"[All Fields] OR "neoplasms"[MeSH Terms] OR "neoplasms"[All Fields] OR "cancer"[All Fields] OR "cancers"[All Fields] OR ("cysts"[MeSH Terms] OR "cysts"[All Fields] OR "cyst"[All Fields] OR "neurofibroma"[MeSH Terms] OR "neurofibroma"[All Fields] OR "neurofibromas"[All Fields] OR "tumor s"[All Fields] OR "tumoral"[All Fields] OR "tumorous"[All Fields] OR "tumour"[All Fields] OR "neoplasms"[MeSH Terms] OR "neoplasms"[All Fields] OR "tumor"[All Fields] OR "tumour s"[All Fields] OR "tumoural"[All Fields] OR "tumourous"[All Fields] OR "tumours"[All Fields] OR "tumors"[All Fields]) OR ("malign"[All Fields] OR "malignance"[All Fields] OR "malignances"[All Fields] OR "malignant"[All Fields] OR "malignants"[All Fields] OR "malignities"[All Fields] OR "malignity"[All Fields] OR "malignization"[All Fields] OR "malignized"[All Fields] OR "maligns"[All Fields] OR "neoplasms"[MeSH Terms] OR "neoplasms"[All Fields] OR "malignancies"[All Fields] OR "malignancy"[All Fields]) OR ("carcinoma"[MeSH Terms] OR "carcinoma"[All Fields] OR "carcinomas"[All Fields] OR "carcinoma s"[All Fields]) OR ("metastasi"[All Fields] OR "neoplasm metastasis"[MeSH Terms] OR ("neoplasm"[All Fields] AND "metastasis"[All Fields]) OR "neoplasm metastasis"[All Fields] OR "metastasis"[All Fields]))  (Direct Oral Anticoagulants OR DOAC OR Novel Oral Anticoagulants OR New Oral Anticoagulants OR NOAC OR Target-specific Oral Anticoagulants OR TOAC OR Direct Factor Xa Inhibitors OR Non-Vitamin K Antagonists OR non-VKA OR Rivaroxaban OR Apixaban OR Edoxaban OR Dabigatran OR Betrixaban OR Vitamin K antagonists OR VKA OR Warfarin OR Coumadin OR Heparin) AND (Cancer OR Tumor OR Malignancy OR Carcinoma OR Metastasis) in Title Abstract Keyword |
| --- | --- |

Supplemental Figure 1: Prisma Flow Diagram


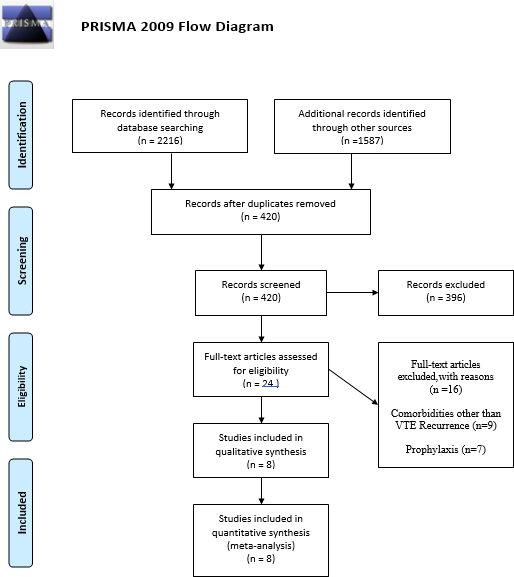


Supplemental Figure 2a. Quality Assessment of Included Randomised Controlled Trials using Cochrane Risk of Bias Tool; Risk of Bias Graph


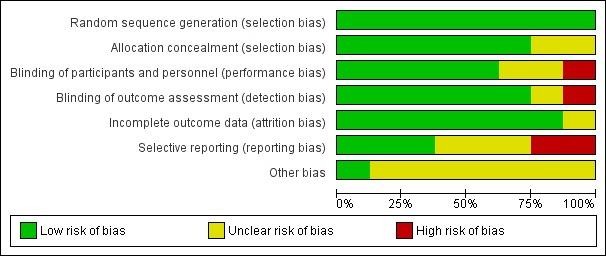


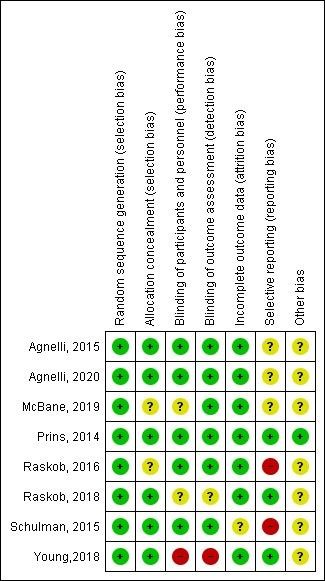
Supplemental Figure 2b. Quality Assessment of Included Randomised Controlled Trials using Cochrane Risk of Bias Tool; Risk of Bias Summary

| **Supplemental Table 2. S2. Baseline Characteristics of Patie**  **Agnelli, 2020** | | | **nts**  **McBane, 2019** | | **Young, 2018** | | **Raskob, 2018** | |
| --- | --- | --- | --- | --- | --- | --- | --- | --- |
|  | Apixaban n = 576 | Dalteparin n = 576 | Apixaba n  n = 150 | Daltepari n  n = 150 | Rivaroxaba n  n = 203 | Daltepari n  n = 203 | Edoxaba n  n = 522 | Daltepari n  n = 524 |
| **Age,y, mean (SD), or (%)** | 67.2±11.3 | 67.2±10.9 | 64.4  (11.3) | 64.0  (10.8) | 67  (median) | 67  (median) | 64.3±11.  0 | 63.7±11.  7 |
| **Female sex no. (%)** | 284(49.3) | 300(52.3) | 78  (52.0) | 77 (51.3) | 87(43) | 105(52) | 245(46.9  ) | 261(49.8  ) |
| **Male sex no. (%)** | 292 (50.7) | 276 (47.7) | 72(48.0) | 73(48.7) | 116(57) | 98(48) | 277  (53.1) | 263  (50.2) |
| **BMI, kg/m2, mean (SD)** | NA | NA | NA | NA | 26.7  (median) | 26.6  (median) | NA | NA |
| **Weight, mean (SD), kg** | 75.7±16.1 | 76.1±16.7 | 84.8  (23.2 | 86.8  (20.5) | NA | NA | 78.8±17.  9 | 79.1±18.  1 |
| **Creatinine clearance ≤50 ml**  **per min — no. (%)** | 51 (8.9) | 61 (10.5) | 14  (9.3%) | 14  (9.3%) | 40 (19) | 38 (18) | 38 (7.3) | 34 (6.5) |
| **Qualifying thrombus n,**  **(%** |  |  |  |  |  |  |  |  |
| **pulmonary embolism only (PE)** | 304 (52.8) | 334 (57.7) | 64  (43.5%) | 57  (38.5%) | 40 (19) | 38 (18) | 328  (62.8) | 329  (62.8) |
| **deep vein thrombosis only (DVT)** | 272 (47.2) | 245 (42.3) | 54  (36.7%) | 52  (35.1%) | 53 (25) | 57 (28) | 194  (37.2) | 195  (37.2) |
| **Type of cancer — no. (%)** |  |  |  |  |  |  |  |  |
| **Active** | 559 (97.0) | 565 (97.6) | NA | NA | NA | NA | 513  (98.3) | 511  (97.5) |
| **History of Cancer** | NA | NA | NA | NA | NA | NA | NA | NA |
| **Recurrent locally advanced**  **or metastatic** | 389 (67.5) | 396 (68.4) | 96  (65.3%) | 97  (66.0%) | 199(98) | 197(97) | NA | NA |
| **Cancer treatment — no.**  **(%)** |  |  |  |  |  |  |  |  |
| **At enrolment** | 350 (60.8) | 367 (63.4) | NA | NA | NA | NA | NA | NA |
| **Withing previous 6mo** | 143 (24.8) | 129 (22.3) | NA | NA | NA | NA | 374  (71.6) | 383  (73.1) |
| **During trial period** | 344 (59.7) | 346 (59.8) | NA | NA | NA | NA | NA | NA |

**Supplemental Table 2. S2. Baseline Characteristics of Patients (Continued)**

| **Raskob, 2016** | | | **Agnelli, 2015** | | **Schulman, 2015** | | **Prins, 2014** | |
| --- | --- | --- | --- | --- | --- | --- | --- | --- |
|  | Edoxaban n = 487 | Warfarin n = 492 | Apixaban n = 274 | Warfarin n = 260 | Dabigatran n = 59 | Warfarin n = 55 | Rivaroxaban n = 491 | Warfarin n = 440 |
| **Age, y, mean (SD), or (%)** | 66.5(62.22) | 66(59.04) | 67.4 | 66.3 | 60.9 ± 13.5 | 65.3 ± 13.2 | 65-75(31.4) | 65-75(39.5) |
| **Female sex no. (%)** | 252(51.7) | 226(45.9) | 133(48.5) | 182(60.0) | 22 (37) | 13 (24) | 219(44.6) | 208(47.3) |
| **Male sex no. (%)** | 235(48.3) | 266(54.1) | 141(51.5) | 78(30.0) | 37(63) | 42(76) | 272(55.4) | 232(52.7) |
| **BMI, kg/m2, mean (SD)** | NA | NA | NA | NA | 28.0 ± 5.4 | 28.2 ± 4.8 | 27.6 (mean) | 27.8(mean) |
| **Weight, mean (SD), kg** | NA | NA | 81.1 | 82.1 | 82.7 ± 16.3 | 82.7 ± 15.6 | NA | NA |
| **Creatinine clearance ≤50 ml per min — no. (%)** | NA | NA | NA | NA | 90.7 ± 27.2 | 83.9 ± 30.4 | 75 | 65 |
| **Qualifying thrombus n, (%** |  |  |  |  |  |  |  |  |
| **pulmonary embolism only (PE)** | 215(44.1) | 229(46.5) | NA | NA | 16 (27) | 8 (15) | NA | NA |
| **deep vein thrombosis only (DVT)** | 272(55.9) | 263(53.5) | NA | NA | 36 (61) | 40 (73) | NA | NA |
| **Type of cancer — no. (%)** |  |  |  |  |  |  |  |  |
| **Active** | 109(22.4) | 99(20.1) | 88 | 81 | NA | NA | NA | NA |
| **History of Cancer** | 378(77.6) | 393(79.9) | 186 | 179 | NA | NA | NA | NA |
| **Recurrent locally advanced or metastatic** | NA | NA | NA | NA | NA | NA | 49(10) | 52(11.8) |
| **Cancer treatment — no. (%)** |  |  |  |  |  |  |  |  |
| **At enrolment** | NA | NA | NA | NA | NA | NA | NA | NA |
| **Withing previous 6mo** | NA | NA | NA | NA | NA | NA | NA | NA |
| **During trial period** | NA | NA | NA | NA | NA | NA | NA | NA |

**Supplemental Figure 3**. Forest displaying subgroup analysis according to type of DOAC for the following outcomes: (A) venous thromboembolism, (B) major bleeding

# Venous thromboembolism


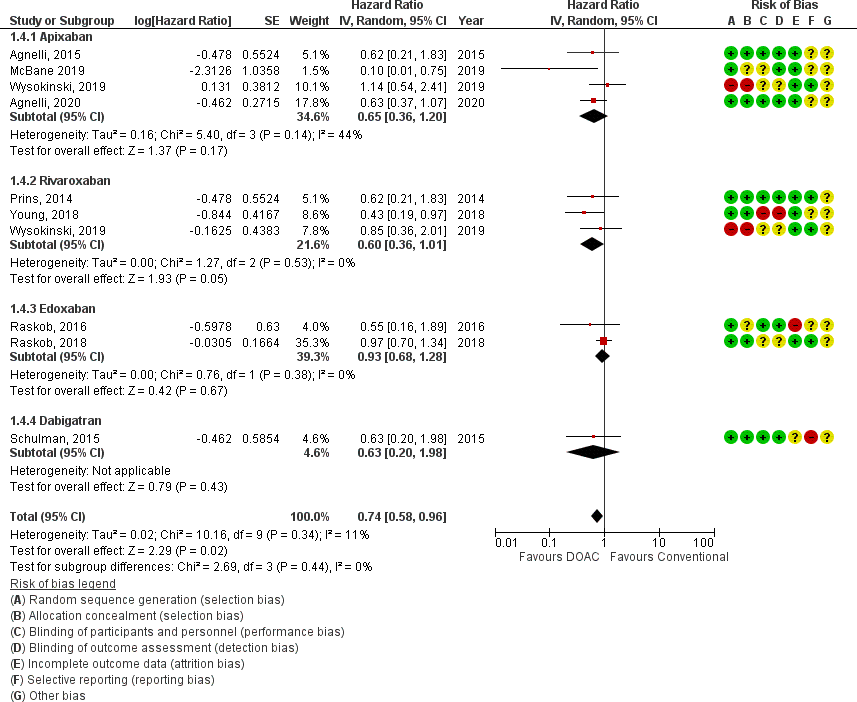


1. **Major Bleeding**


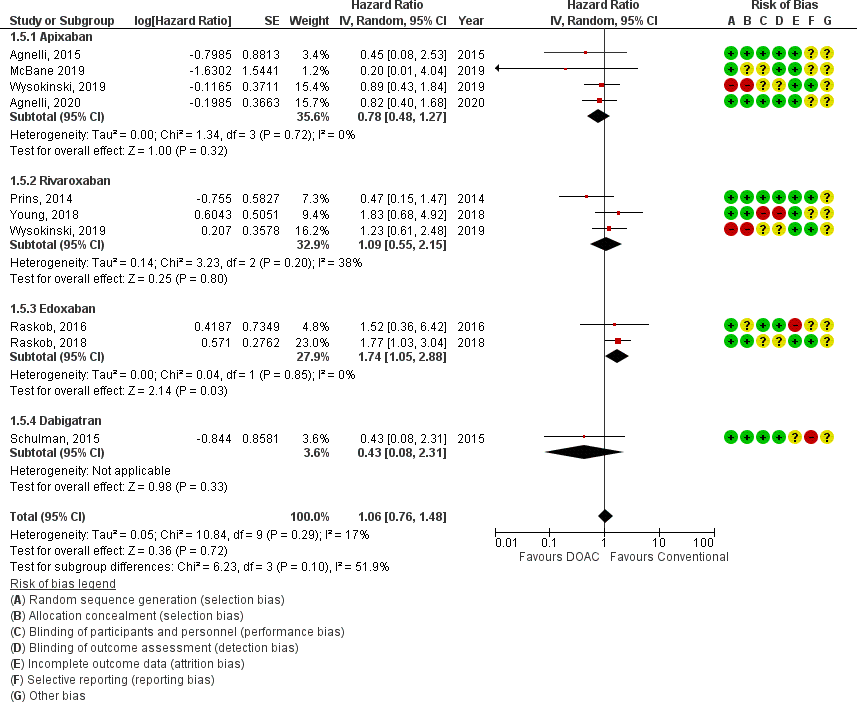


**Supplemental Figure 4**. Forest plot displaying subgroup analysis according to type of cancer for venous thromboembolism


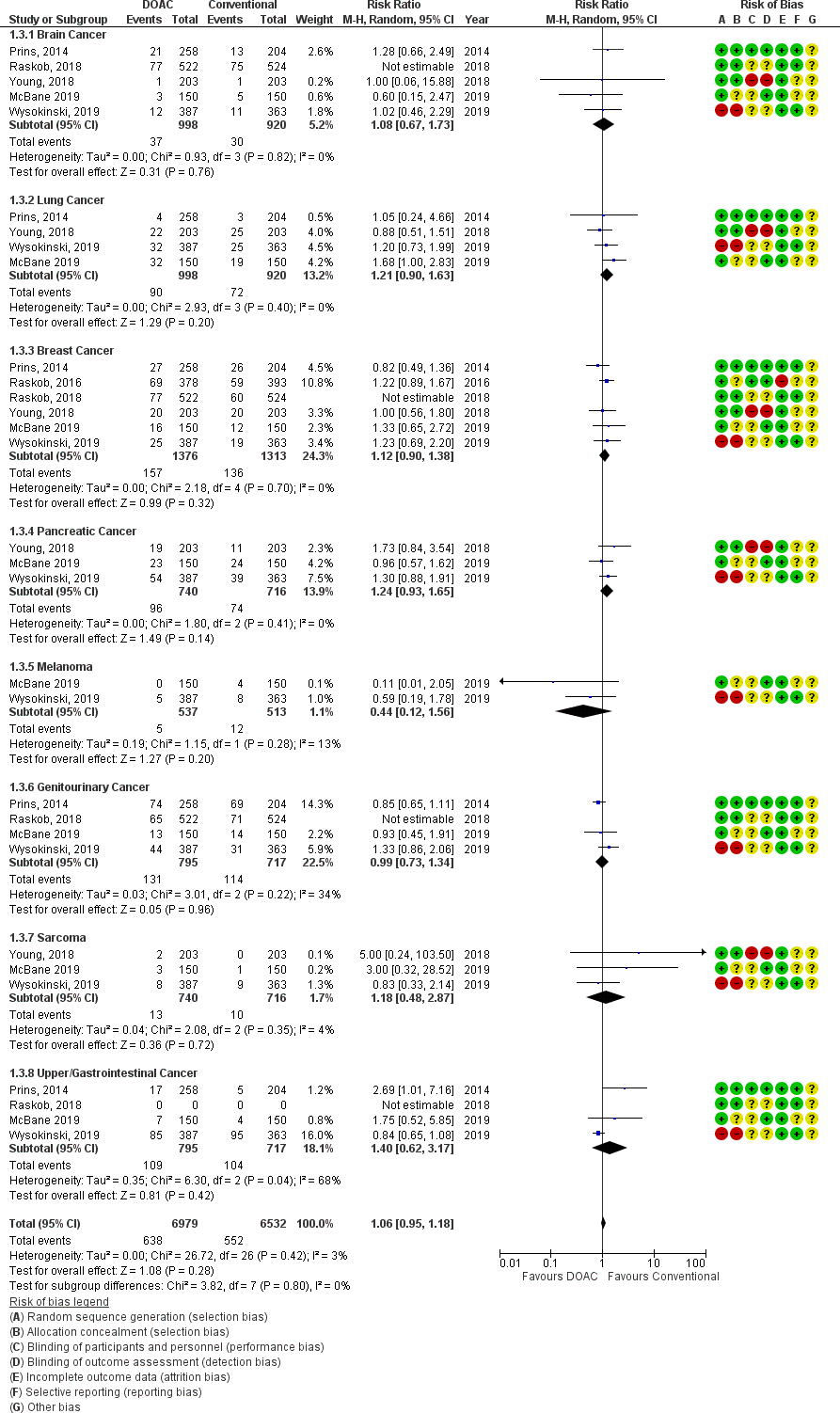


**Supplemental Figure 5**. Forest displaying sensitivity analysis for clinically relevant non- major bleeding


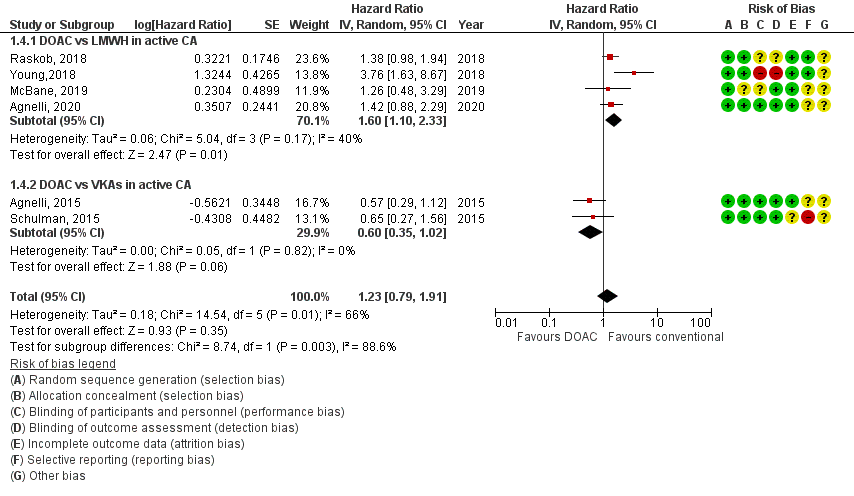

Supplement: Multimedia component 1 [file mmc1.docx]
